# Quality of Life in Neurotypical Siblings of Children with an Autism Spectrum Disorder: Associations with Parental Social Support and demographic variables

**DOI:** 10.1192/j.eurpsy.2022.1136

**Published:** 2022-09-01

**Authors:** E. Koukouriki

**Affiliations:** University of Ioannina, Special Education Lab, Department Of Primary Education, Ioannina, Greece

**Keywords:** Quality of Life, neurotypical siblings, parental perceived social support, Autism Spectrum Disorders

## Abstract

**Introduction:**

Health Related Quality of Life (HRQOL) is acknowledged as an important construct in describing the individuals overall condition within the health context. In the case of families with Autism Spectrum Disorders (ASD), neurotypical siblings’ HRQOL is an important concept for this at-risk population. Social support has been identified as an important protective factor against parental psychological problems. However, possible associations between HRQOL in neurotypical ASD-siblings and parental variables, such as perceived social support, remained unexplored in the ASD-siblings’ literature.

**Objectives:**

This study aimed to investigate any association between HRQOL in neurotypical ASD-siblings and parental perceived social support and key demographic variables.

**Methods:**

118 parent-child-dyads from Greek ASD-families that fulfilled inclusion criteria participated in this study and answered a demographic questionnaire. Parents were administered the Multidimensional Scale of Perceived-Social-Support (MSPSS), while the children answered the Kidscreen-27. A hierarchical multiple regression was performed to test the hypothesis.

**Results:**

Hierarchical regression showed that neurotypical siblings HRQOL (as measured with KIDSCREEN-27 total score) was associated with perceived social support from the family (std beta=0.184; p=0.027; model 1), and this association persisted after demographics entered the model (std beta=0.369; p=0.015; model 2). Thus, it was found that the sibling’s HRQOL was associated with perceived social support from the family independent of demographics.

**Conclusions:**

The results of the present study showed that the HRQOL in ASD-siblings is associated with perceived social support from the family, pointing to the need for supporting siblings and designing effective interventions in order to prevent possible mental health problems in neurotypical siblings of ASD individuals in the future.

**Disclosure:**

No significant relationships.

